# A double-edged sword: CRISPR-Cas9 is emerging as a revolutionary technique for genome editing

**DOI:** 10.1186/s40779-015-0054-1

**Published:** 2015-10-15

**Authors:** Chun-xiao Li, Hai-li Qian

**Affiliations:** State Key Laboratory of Molecular Oncology, Cancer Hospital, Chinese Academy of Medical Sciences, Beijing, 200021 China

**Keywords:** CRISPR-Cas9, Genomic engineering, Ethical controversy

## Abstract

In May 2015, Professor Xiao Yang authored a review on the development of CRISPR-Cas9 techniques in the journal of *Military Medical Research*. This review provided a valuable overview of this major scientific advance. It has been four years since the first publication of the CRISPR-Cas9 breakthrough (Science. 2012; 337: 816–21). The use of this technique has expanded into various scientific areas and is being developed into a systematic technical platform that may contribute to many bioengineering fields involving DNA sequence editing.

## Correspondence/findings

Dear editor,

In May 2015, Professor Xiao Yang authored a review on the development of CRISPR-Cas9 technique in the journal of *Military Medical Research* [1]. This review provided a valuable overview of this major scientific advance. It has been four years since the first publication of the CRISPR-Cas9 breakthrough [2]. The use of this technique has expanded into various scientific areas and is being developed into a systematic technical platform that may contribute to many bioengineering fields involving DNA sequence editing.

The advantages of the CRISPR(Clustered Regularly Interspaced Short Palindromic Repeats)-Cas9 technology include its economy, high efficiency, precise targeting and flexible technical extension compared with traditional DNA sequence modifying measures such as transcription activator-like effector nuclease (TALEN,transcription activator-like (TAL) effector nucleases) [3]. The published literature has shown the utility of CRISPR-Cas9 in both DNA sequence knock-in and knock-out contexts. The alterations can range from single nucleotide editing to the modification of multiple genome-wide genomic sites [4, 5]. It is easy to delete genes in cells or to create genetically modified karyotypes [6]. The CRISPR-Cas-9 strategy is a convenient method of screening functional genes in life processes and disease development. The technology may also be used as a potential “surgical knife” to correct genomic mutations or create new creatures by changing the inherited phenotypes. In addition to the genomic engineering applications that professor Xiao Yang mentioned, the protospacer adjacent motif (PAM) sequence-limited specificity of the CRISPR-Cas9 system has been used to circumvent engineering Cas9 derivatives. This property provides flexibility to CRISPR-Cas9 targeting strategies [7]. Poulami et al. also found another aspect of CRISPR-mediated immunity. The authors found the Type III CRISPR-Cas immune system was able to cleave DNA and RNA during infection [8]. The technique of CRISPR-Cas9 is still in development. If nonhomologous end joining activity is inhibited in vivo, then the efficiency of precise genome editing with CRISPR-Cas9 can be substantially increased [9].

The CRISPR-Cas9 technology does have limitations associated with targeting ability. Several off-target mutations have been detected by genome-sequencing due to its high specificity. This prohibits its potential use in correcting disease-associated mutations [10]. The rapidly expanding application of the CRISPR-Cas9 technique also creates an ethical controversy because it may be used to manipulate human germ cells. Manipulating human germ cells using CRISPR-Cas9 does not present technical obstacles. However, its potential off-target effects must be considered when a genetically modified individual is created with this tool. Each additional step forward will further improve the technology. Therefore, this technique should be used cautiously before modifying human inheritance. A recent publication in the journal Protein & Cell by Junjiu Huang’s group in China revealed the first attempt to modify human tripronuclear zygotes [11]. This report caused a fierce debate regarding whether this research is breaking the ethical ban on modifying the human germ cell genome [12, 13]. Simultaneously, a project led by George Church at Harvard University tried to correct genomic BRCA1(BREAST CANCER 1) mutations to decrease the risk of breast cancer in the next generation. This study was suspended indefinitely. In addition to the ethical concerns, there are technical concerns to address. The current CRISPR-Cas9 technique is not sufficiently mature to adjust human inheritance. The first issue is that CRISPR-Cas9 induces off-target changes to the genome. The second possible issue is that not all of the functions of the candidate gene are fully understood. Therefore, we cannot appreciate the consequences of genome editing in offspring.

While we are joyfully celebrating the progress brought to science by the development of CRISPR-Cas9, we must use caution in applying this technology.
